# Supplementary material for: Gene editing and elimination of latent herpes simplex virus in vivo
Source: Nat Commun. 2020 Aug 18;11:4148. doi: 10.1038/s41467-020-17936-5 (PMC7435201; doi:10.1038/s41467-020-17936-5)
Supplement: Supplementary file 1 — Supplementary Information [file 41467_2020_17936_MOESM1_ESM.pdf]

# Gene editing and elimination of latent herpes simplex virus in vivo

Aubert et al.

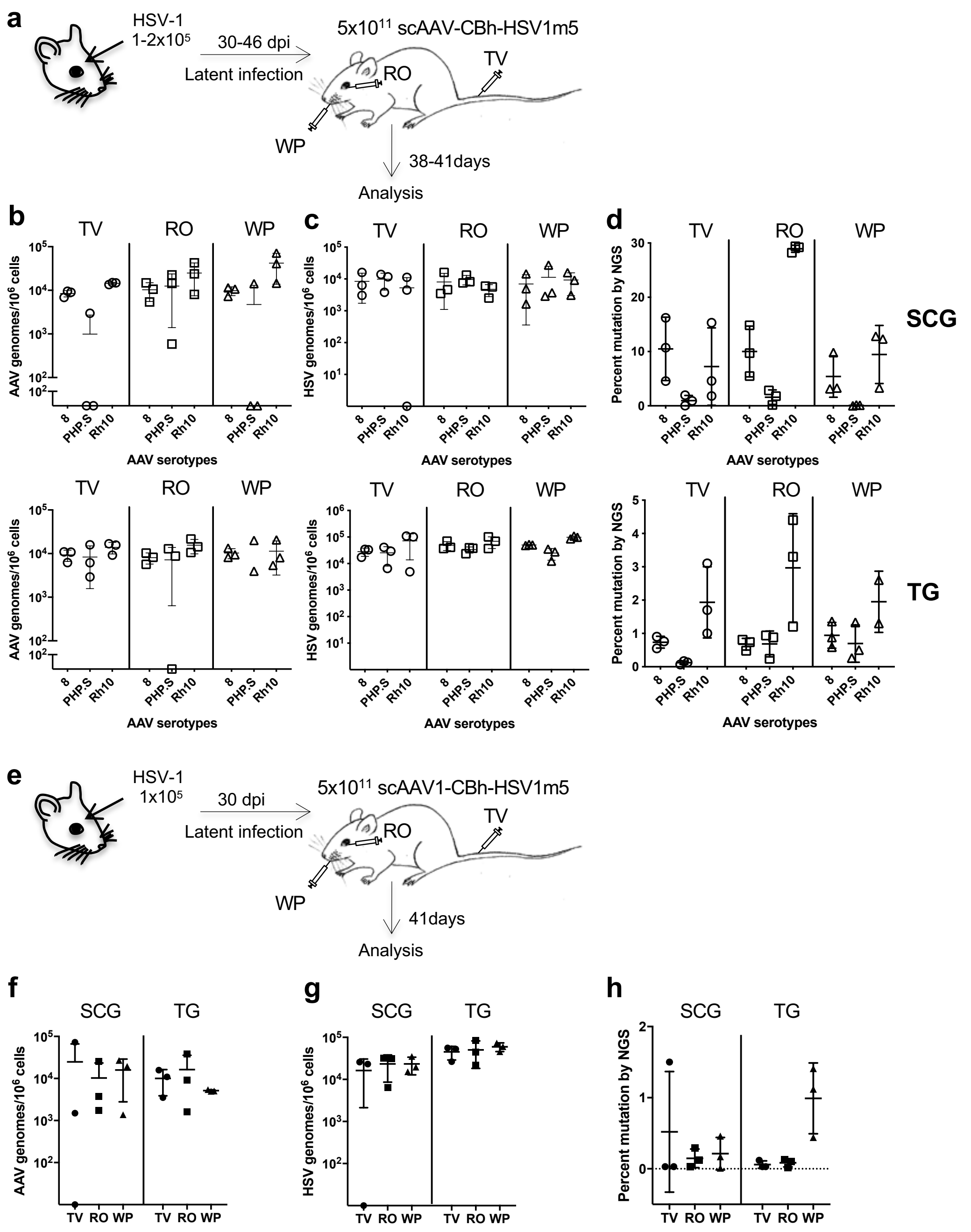


**Supplementary Figure 1**. **Screening of AAV serotypes and route of administration for nuclease delivery to ganglionic neurons. a**, Mice were infected with 1-2 x $10^5$ PFU HSV-1 17+ in the right eye following corneal scarification, and 30-46 days later after HSV latency establishment, were administered 5 x $10^{11}$ vgs scAAV-CBh-HSV1m5 packaged with AAV serotype 8, PHP.S or Rh10 by either tail vein (TV, circles, n = 3), retro-orbital (RO, squares, n = 3), or whisker pad (WP, triangles, n = 3) injection. Analysis was performed 38-41 days later. **b**, AAV and **c**, HSV genomes from right ipsilateral SCG (top panels) or TG (bottom panels) were quantified by ddPCR. **d**, Mutagenic events at the HSV1m5 target site in HSV genomes in SCG (top panel) or TG (bottom panel) were quantified by NGS analysis. **e**, Mice infected with $10^5$ PFU HSV-1 17+ in the right eye following corneal scarification for 30 days, were administered 5 x $10^{11}$ vgs scAAV1-CBh-HSV1m5 by either TV (circles, n = 3), RO (squares, n = 3) or WP (triangles, n = 3) injection. At 41 days post AAV administration, ipsilateral SCG and TG were collected for analysis. **f**, AAV and **g**, HSV genomes from SCG and TG were quantified by ddPCR. **h**, mutagenic events at the HSV1m5 target site in HSV genomes in SCG and TG were quantified by NGS analysis. All data are presented as mean values +/- SD. Source data are provided as a Source Data file.

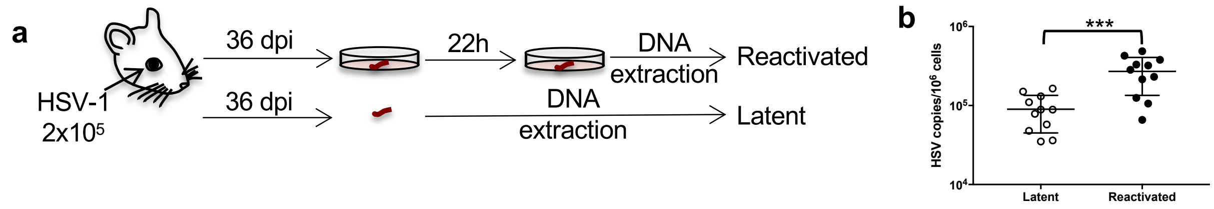


**Supplementary Figure 2**. **HSV loads after tissue explant reactivation. a**, Mice were infected with 2 x $10^5$ PFU HSV-1 17+ in the right eye following corneal scarification, and 36 days later right ipsilateral TG was collected and total DNA extracted either immediately or after being subjected to TG explant reactivation by placing them into culture media for 22h. **b**, ddPCR quantification of HSV genomes from latent infected TG (open circles, n = 11) or reactivated TG (closed circles, n = 11). One-tail unpaired *t-test* shows a significant 3-fold increase in viral loads from reactivated TG compared with latent TG, $p = 0.0002$. * $p < 0.05$, ** $p < 0.01$, *** $p < 0.001$, **** $p < 0.001$ significantly different from controls. All data are presented as mean values +/- SD. Source data are provided as a Source Data file.

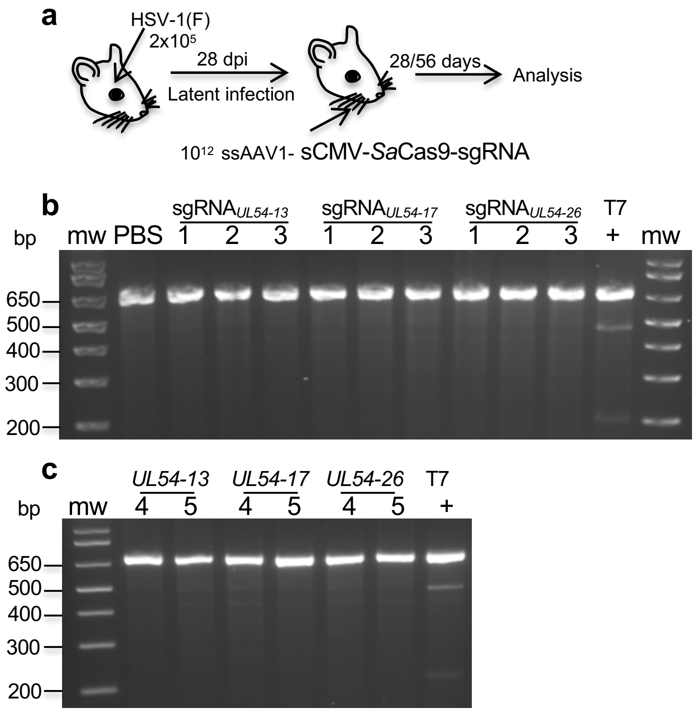


**Supplementary Figure 3. T7E1 analysis of CRISPR/Cas9 gene editing of latent HSV *in vivo*. a,** Mice latently infected with 2 x $10^5$ PFU HSV-1(F) for 28 days were injected in the right whisker pad with $10^{12}$ vgs ssAAV1-sCMV-*Sa*Cas9-sgRNA$_{UL54}$ (n=5 per sgRNA$_{UL54}$). Analysis was performed at either 28 (n=3 mice per sgRNA$_{UL54}$) or 56 (n=2 mice per sgRNA$_{UL54}$) days after AAV injection. **b-c,** Detection of mutations by T7E1 assay in HSV genomes from TG DNA of treated mice from the experiment described in Figure 5d-f, and collected at either **b**, 28 or **c**, 56 days after AAV administration. Source data are provided as a Source Data file.

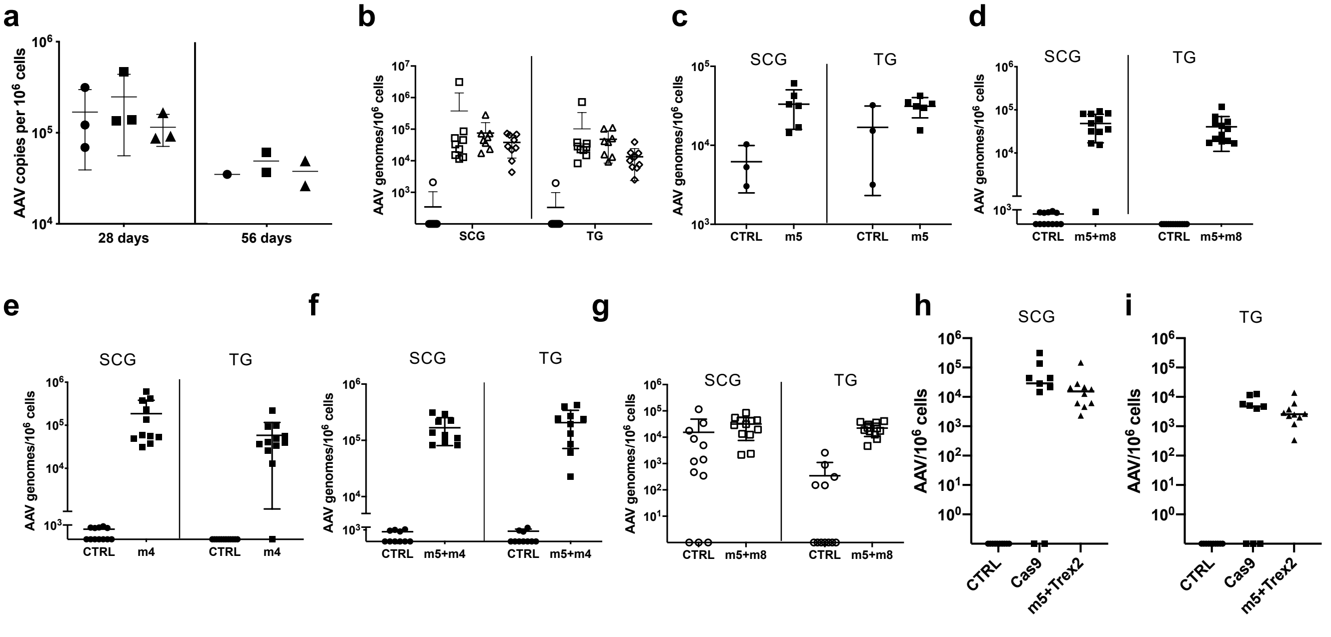


**Supplementary Figure 4. ddPCR quantification of AAV genomes in ganglia. a**, Levels of AAV transduction in TG from infected mice injected in the right whisker pad with $10^{12}$ vgs ssAAV1-sCMV-*Sa*Cas9-sgRNA$_{UL54}$ (n = 5 per sgRNA$_{UL54}$). sgRNA$_{UL54\text{-}13}$ (circles), sgRNA$_{UL54\text{-}17}$ (squares), and sgRNA$_{UL54\text{-}26}$ (triangles). Analysis was performed at either 28 (n = 3 mice per sgRNA$_{UL54}$) or 56 (n = 2 mice per sgRNA$_{UL54}$) days post AAV exposure (see Fig. 5d-f). **b**, Levels of AAV transduction of SCG and ipsilateral TG of latently infected mice left untreated (Controls, circles n = 8) or administered by whisker pad injection either 5 x $10^{11}$ vgs scAAV8-CBh-m5 (squares, n = 9), 5 x $10^{11}$ vgs scAAV8-CBh-m8 (triangles, n = 8) or 5 x $10^{11}$ vgs of each scAAV8-CBh-m5 and scAAV8-CBh-m8 (diamonds, n = 9). Analysis was performed 31 days later (see Fig. 1). **c**, Levels of AAV transduction of SCG and ipsilateral TG of latently infected mice either left untreated (CTRL, circles n = 3) or administered 0.5-1x$10^{12}$ scAAV-Rh10-CBh-m5 by RO injection (m5, squares n = 6). Analysis was performed 41 days later (see Fig. 2a-c). **d**, Levels of AAV transduction of SCG and ipsilateral TG of latently infected mice either left untreated (CTRL, circles n = 12) or administered by RO injection 5x$10^{11}$ vgs scAAV-Rh10-CBh-m5 + 5x$10^{11}$ vgs scAAV-Rh10-CBh-m8 (m5+m8, squares, n = 12). Analysis was performed 33-35 days later (see Fig. 2d-f). **e**, Levels of AAV transduction of SCG and ipsilateral TG of latently infected mice either left untreated (CTRL, circles n = 12) or administered by RO injection 1x$10^{12}$ vgs scAAV-Rh10-CBh-m4 (m4, n = 12). Analysis was performed 33-35 days later (see Fig. 3a-c). **f**, Levels of AAV transduction of SCG and ipsilateral TG of latently infected mice either left untreated (CTRL, circles n = 10) or administered by RO injection 5x$10^{11}$ vgs scAAV-Rh10-CBh-m5 + 5x$10^{11}$ vgs scAAV-Rh10-CBh-m4 (m5+m4, squares, n = 10). Analysis was performed 33-35 days later (see Fig. 3d-f). **g**, Levels of AAV transduction of reactivated SCG and ipsilateral TG of latently infected mice either left untreated (CTRL, circles n = 12) or administered 5x$10^{11}$ vgs scAAV-Rh10-CBh-m5 + 5x$10^{11}$ vgs scAAV-Rh10-CBh-m8 (m5+m8, squares, n = 12) by RO injection (see Fig. 4). **h-i** Levels of AAV transduction of SCG (**h**) and ipsilateral TG (**i**) of latently infected mice either left untreated (CTRL, circles n = 10) or administered by RO injection either dual sgRNA therapy consisting of $10^{12}$ vgs ssAAVRh10-sCMV-*Sa*Cas9-sgRNA$_{UL54\text{-}26}$ and $10^{12}$ vgs ssAAVRh10-sCMV-*Sa*Cas9-sgRNA$_{UL30\text{-}10}$ (Cas9 squares, n=10) or meganuclease therapy of $10^{12}$ vgs ssAAVRh10-smCBA-m5-Trex2-mCherry (m5+Trex2 triangles, n=10). Analysis was performed 28/29 days later (see Fig. 6). All data are presented as mean values +/- SD. Source data are provided as a Source Data file.

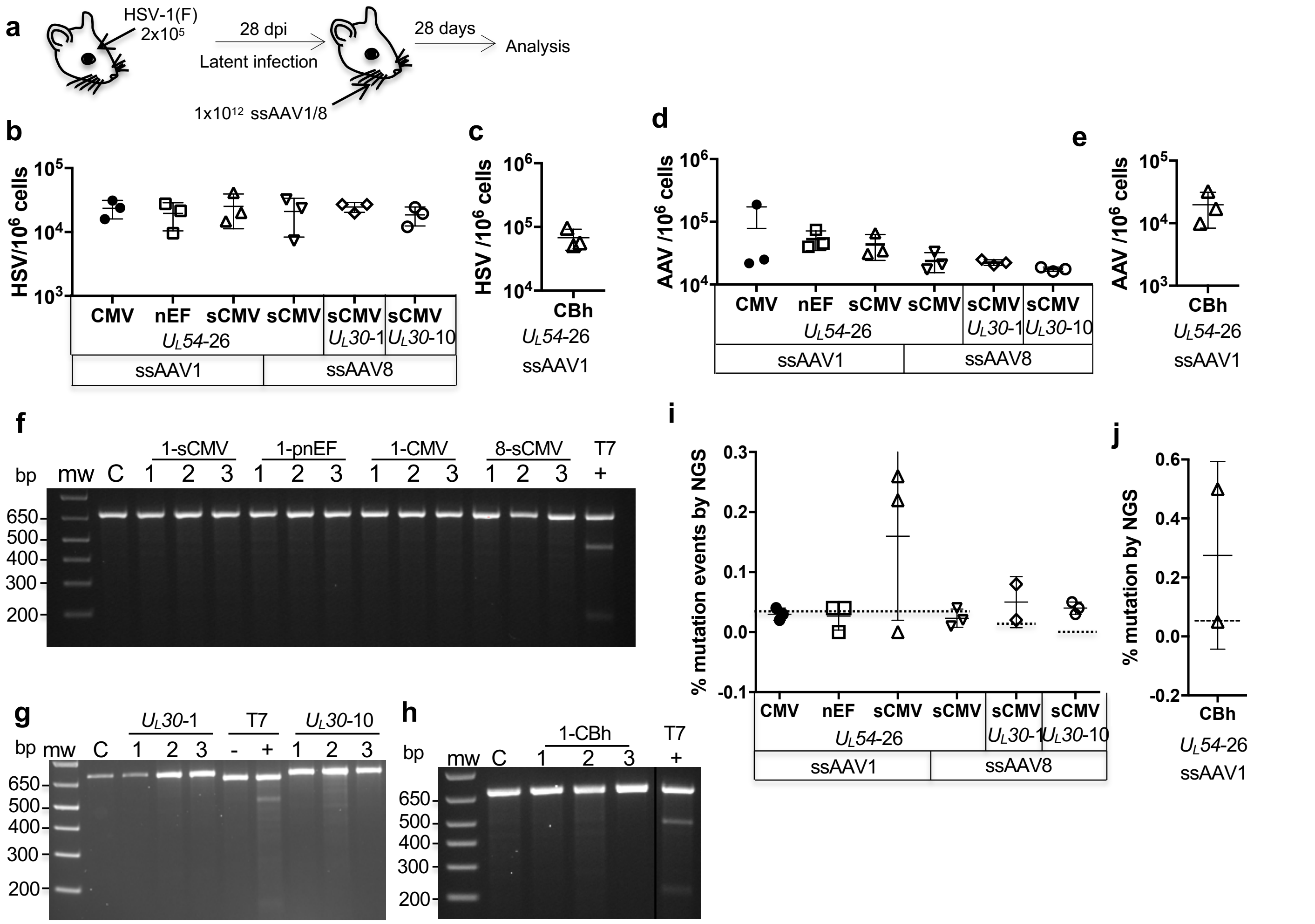


**Supplementary Figure 5. Poor efficiency of HSV gene editing by *Sa*Cas9 in infected mice. a**, Mice were latently infected with 2 x $10^5$ PFU HSV-1(F) for 28 days, and then injected in the right whisker pad (n = 3) with $10^{12}$ vgs *Sa*Cas9-expressing ssAAV1 or ssAAV8 under the indicated promoter and sgRNA targeting either the $U_L54$ ($U_L54$-26: sgRNA$_{UL54-26}$) or $U_L30$ ($U_L30$-1: sgRNA$_{UL30-1}$ and $U_L30$-10: sgRNA$_{UL30-10}$) genes (n = 3 per group). Analysis was performed at 28 days after AAV exposure. **b-c**, Levels of HSV and **d-e**, levels of AAV genomes were quantified by ddPCR in right (ipsilateral) TGs from infected mice. **f-h,** Detection of mutations by T7E1 assay in HSV genomes from TG DNA 28 days after injection with **f,** ssAAV1 (1) or ssAAV8 (8) carrying *Sa*Cas9 under the indicated promoter and sgRNA$_{UL54-26}$ ; **g**, ssAAV8 carrying *Sa*Cas9 under the sCMV promoter, along with the indicated sgRNA$_{UL30}$; **h**, ssAAV1 (1) carrying *Sa*Cas9 under the CBh promoter along with sgRNA$_{UL54-26}$. T7-: T7E1 negative control; T7+: T7E1 positive control, mw: molecular weight marker, bp: base pairs. The gel images were cropped. **i-j**, Mutagenic event detection by NGS analysis of the PCR products used in the T7E1 analysis. The dotted lines mark the levels of background mutation detected at the site targeted by the respective sgRNA in PBS treated animals. All data are presented as mean values +/- SD. Source data are provided as a Source Data file.

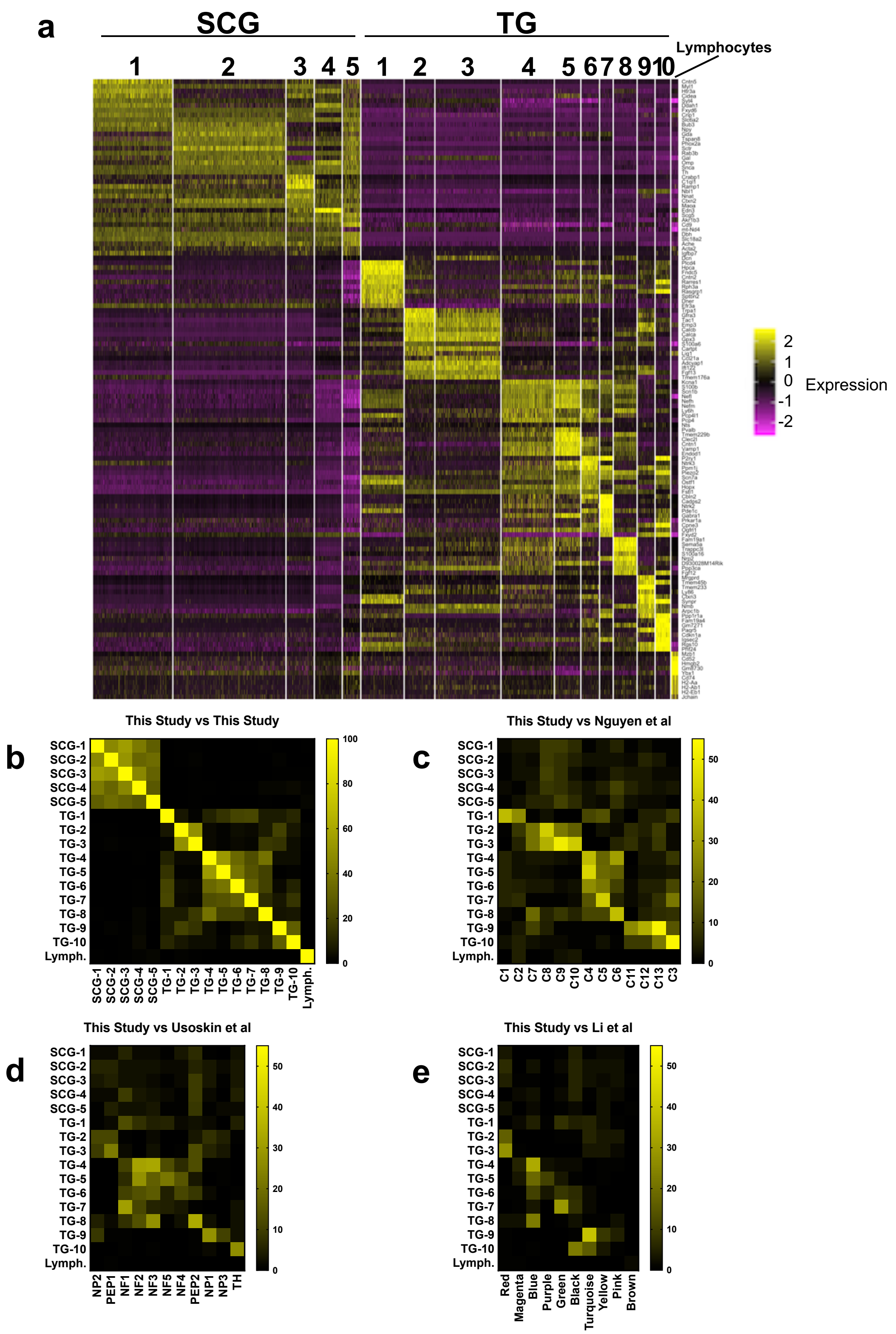


**Supplementary Figure 6. Gene expression patterns that define cluster identity. a,** Heatmap of the top 10 most upregulated genes in each cluster. **b-e**, Heatmaps generated by comparing the top 100 most upregulated genes from each cluster of our study with to the top 100 most upregulated genes from the cluster of either **b**, our study, **c**, Nguyen et al.[30], **d**, Usokin et al.[28], **e**, Li et al.[29]. The color scale indicates the percent of overlapping genes.

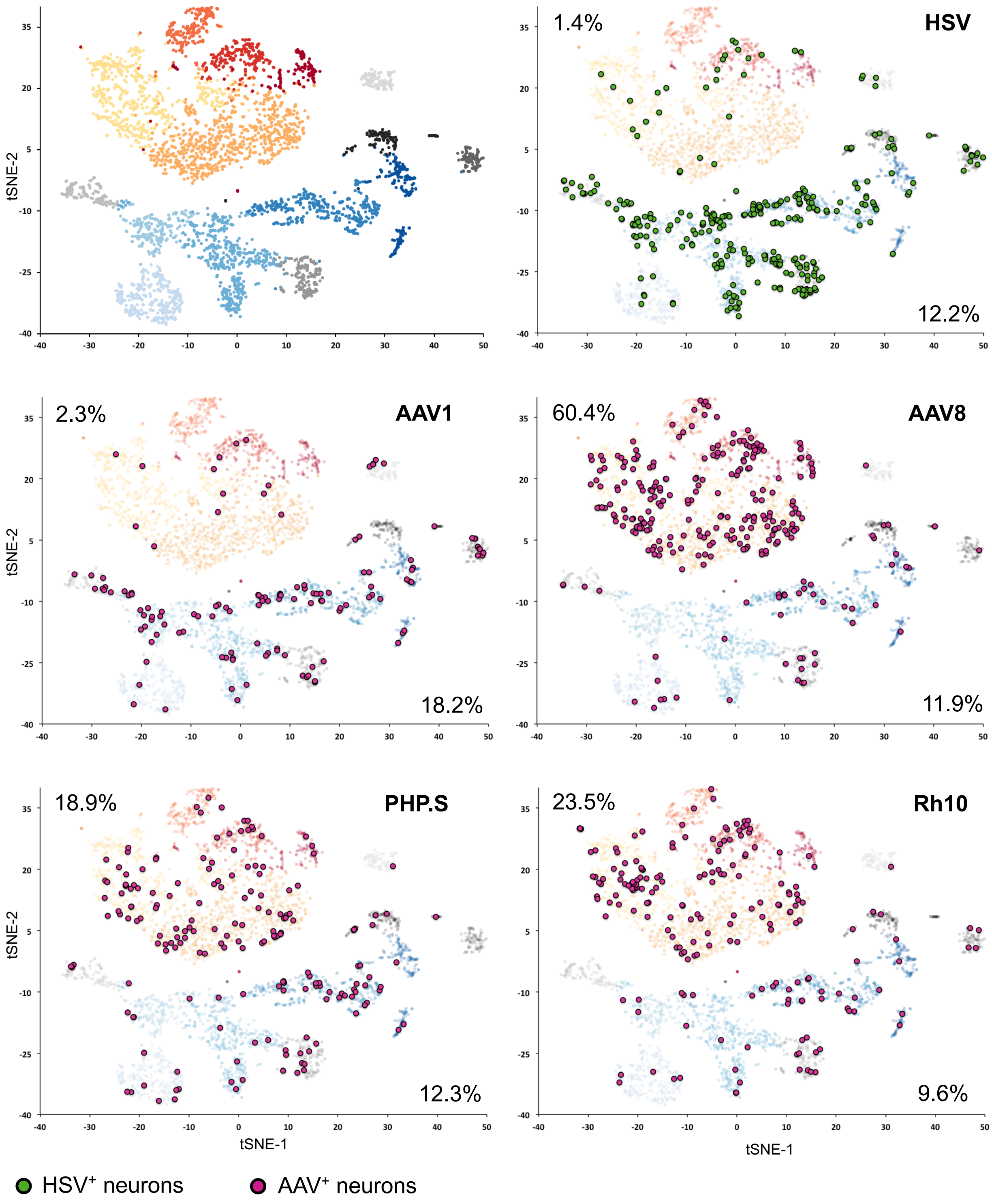


*Note that ¼ of the mice received each of the 4 AAV serotypes, and samples were pooled for library construction and sequencing. Therefore, each serotype could transduce a theoretical maximum of ¼ of the neurons. All mice were infected with HSV.

**Supplementary Figure 7. Distribution of HSV and AAV positive cells across clusters.** Neurons that express HSV genes (green dots) or the transgene delivered via the indicated AAV (magenta dots) were overlaid onto tSNE plots. Note that only one-quarter of the analyzed animals received each AAV serotype, so the actual saturation of neuronal subsets is greater than it appears in this representation. All animals received HSV. Total percentage of transgene positive neurons from SCG and TG is indicated in the upper left and lower right, respectively.

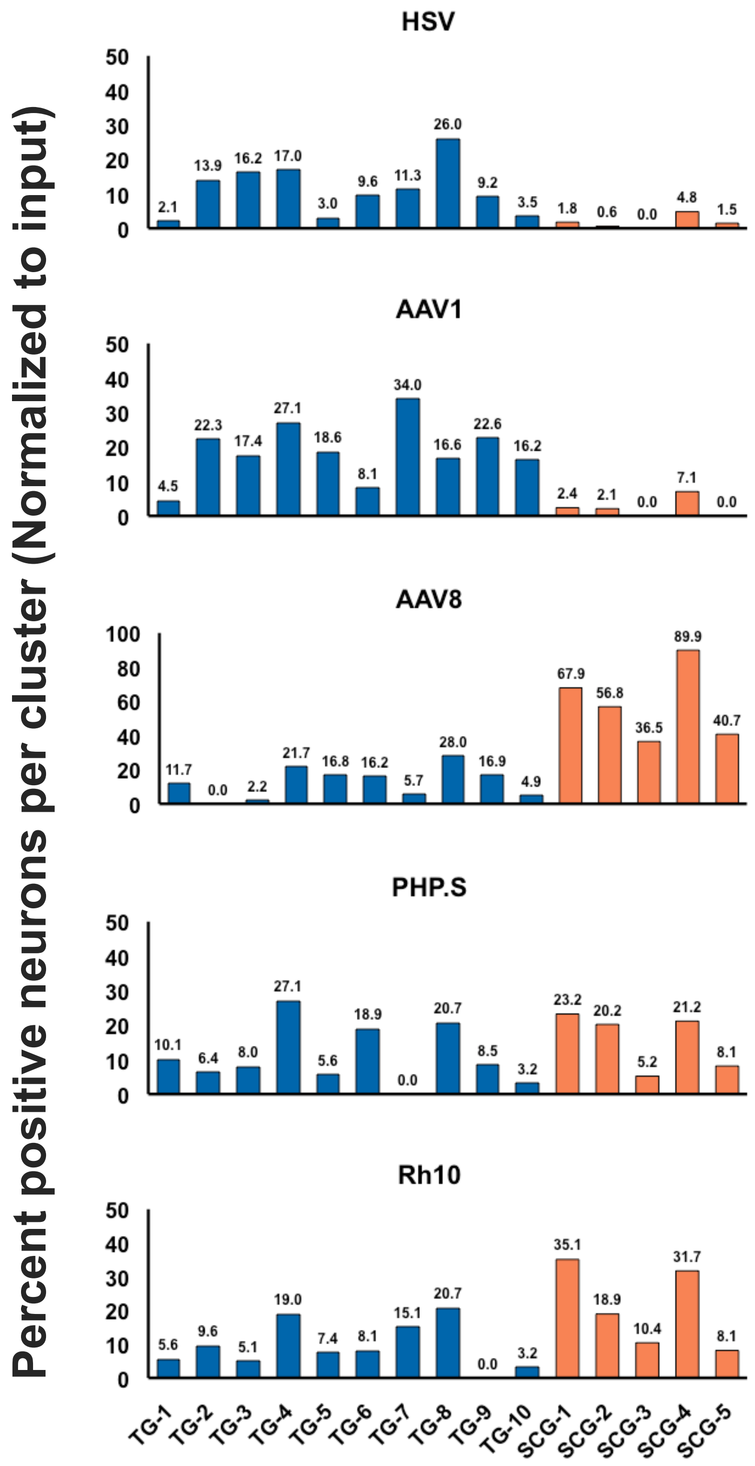


**Supplementary Figure 8. Percentage of neurons in each neuronal cluster positive for HSV or the indicated AAV transgene.** TG-1 to TG-10 clusters (blue bars) and SCG-1 to SCG-5 clusters (orange bars). For AAV expression, percentages were normalized to input as described in Methods.

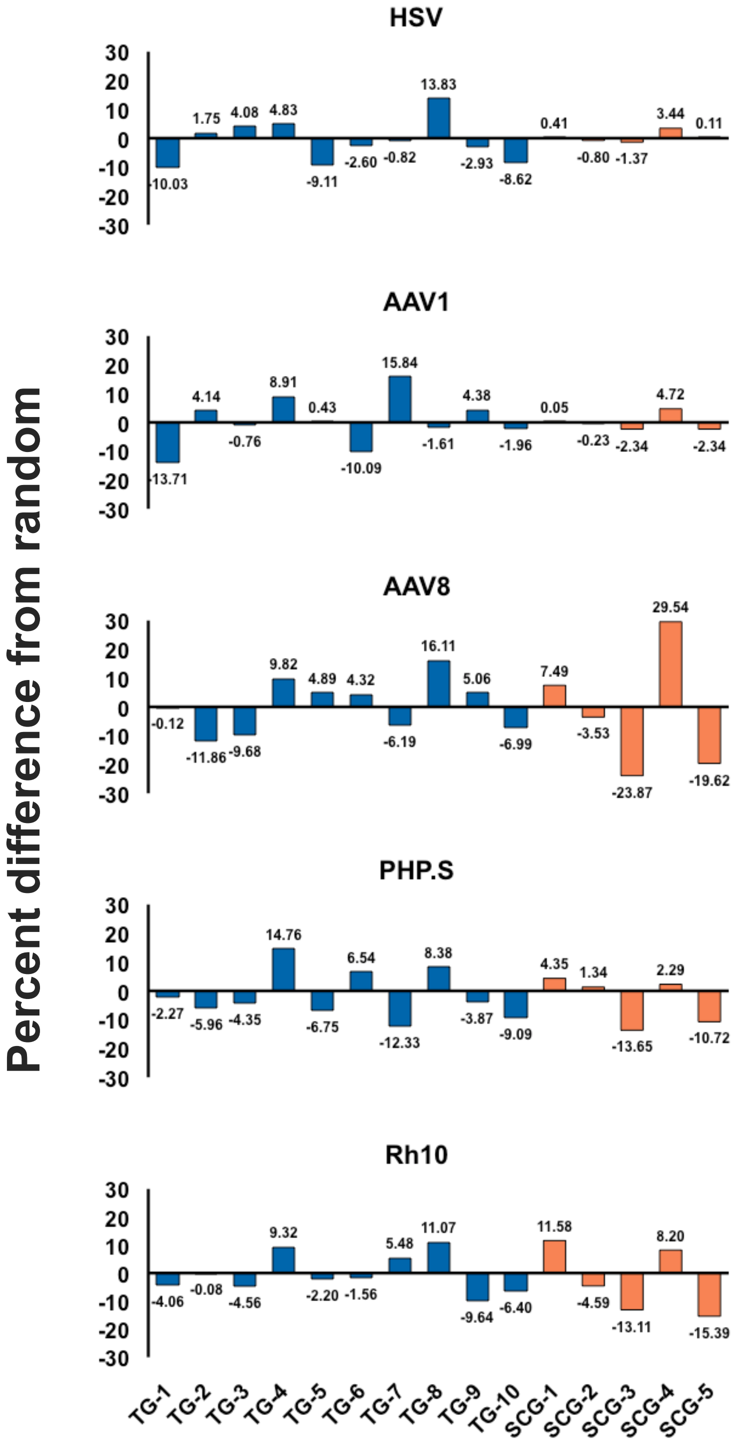


**Supplementary Figure 9. Percent difference in the fractional distributions of HSV+ or AAV+ cells within clusters relative to a random distribution.** TG-1 to TG-10 clusters (blue bars) and SCG-1 to SCG-5 clusters (orange bars). The following formulas were used to compute the percent difference from random: For HSV+ cells: Percent difference from random = [(Number of HSV+ cells detected in the cluster - expected number of HSV+ cells in the cluster[1])/total number of cells in the cluster] x 100. For AAV+ cells: Percent difference from random = [(Number of AAV+ cells detected in the cluster - expected number of AAV+ cells in the cluster[1])/Normalized total number of cells in the cluster[2]] x 100.

[1]The expected number of positive cells in the cluster = number of positive cells within a tissue (TG or SCG) x [total number of cells in the cluster/total number of cells in tissue].

[2]The normalized total number of cells in the cluster = the total number of cells in the cluster x the fraction of cells contributed by mice injected with a given AAV serotype.

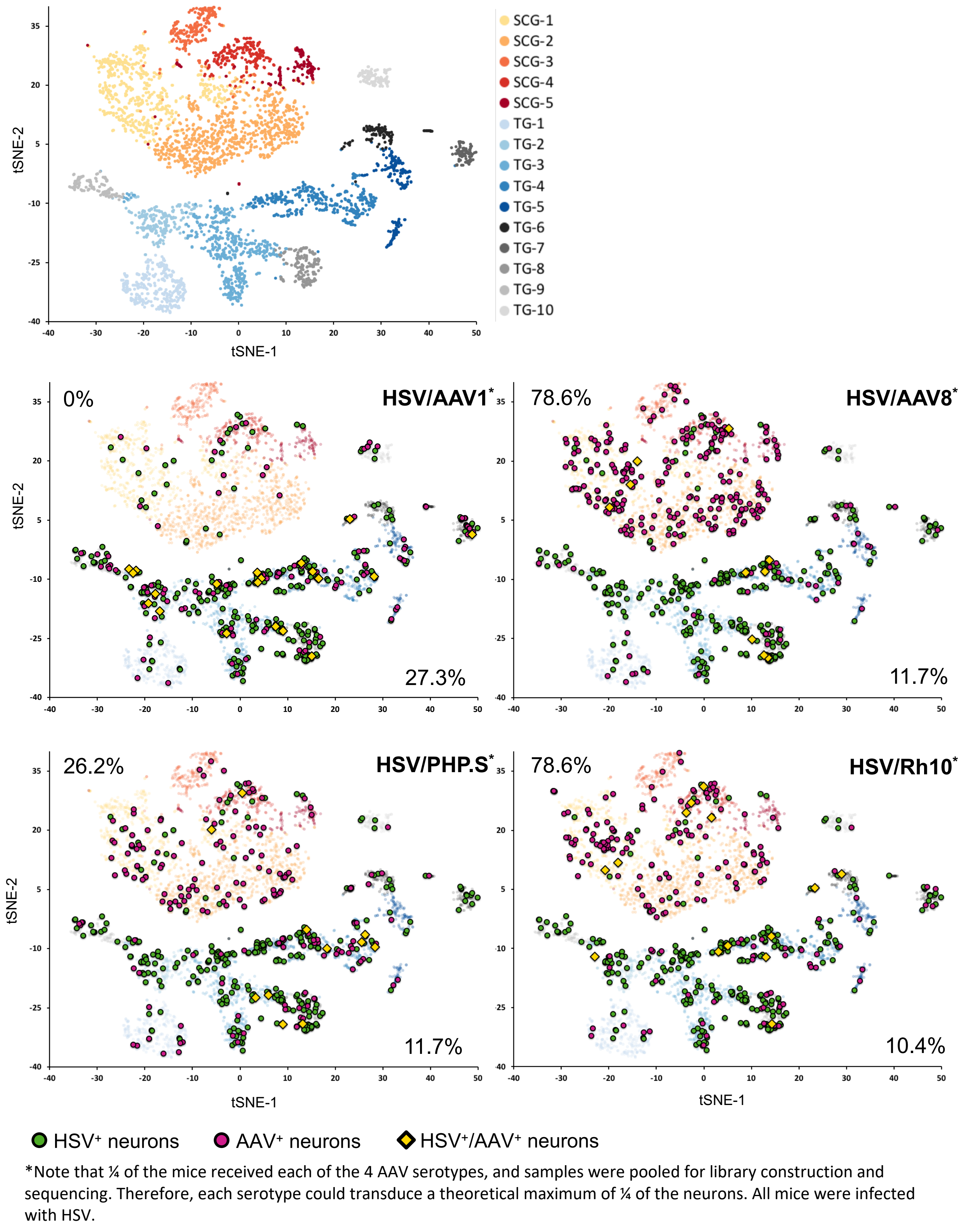


**Supplementary Figure 10. Distribution of HSV and AAV double positive cells across clusters.** Cells that express an HSV gene and the transgene delivered via the indicated AAV were overlaid onto the tSNE plot. Neurons positive for HSV transcript HSV+ (green dots), the transgene transcript of the indicated AAV serotype (magenta dots) or both HSV+/AAV+ (yellow dots). Only one-quarter of the analyzed animals received each AAV serotype, so the actual saturation of neuronal subsets is greater than it appears in this representation. All animals received HSV. Percentage of HSV positive neurons also positive for AAV transgene in SCG and TG is indicated in the upper left and lower right, respectively.

**Supplementary Table 1**. NGS analysis of CRISPR/Cas9 induced mutations in HSV genomes from infected neuronal cultures treated with *Sa*Cas9 and sgRNA$_{UL54\text{-}26}$

| *Target site sequence for sgRNA$_{UL54\text{-}26}$*[1] | *Mutation* | *Frequency* | *Percent* |
|---|---|---|---|
| CATGGCCTTGGCGGTCGATGCGGCCC**GAGGAT**TGCCGGC | wild type | 3490 | 50.87 |
| CATGGCCTTGGCGGTCGATGCGGC--**GAGGAT**TGCCGGC | -2 | 1675 | 24.41 |
| CATGGCCTTGGCGGTCG------CCC**GAGGAT**TGCCGGC | -6 | 186 | 2.71 |
| CATGGCCTTGGCGGTCGATGCGG----**AGGAT**TGCCGGC | -4 | 143 | 2.08 |
| CATGGCCTTGGCGGTCGAT----CCC**GAGGAT**TGCCGGC | -4 | 143 | 2.08 |
| CATGGCCTTGGCGGTCGA----------**GGAT**TGCCGGC | -10 | 122 | 1.78 |
| CATGGCCTTGGCGGTCGATGC---CC**GAGGAT**TGCCGGC | -3 | 120 | 1.75 |
| CATGGCCTTGGCGGTCGATGCGGCC-**GAGGAT**TGCCGGC | -1 | 98 | 1.43 |
| CATGGCCTTGGCGGTCGATGC--CCC**GAGGAT**TGCCGGC | -2 | 89 | 1.30 |
| CATGGCCTTGGCGGTCGATGCGG--------**T**TGCCGGC | -8 | 87 | 1.27 |
| CATGGCCTTGGCGGTCGATGCG-----**AGGAT**TGCCGGC | -5 | 80 | 1.17 |
| CATGGCCTTGGCGGTCGATGCG-CCC**GAGGAT**TGCCGGC | -1 | 79 | 1.15 |
| CATGGCCTTGGCGGTCGATGCG---C**GAGGAT**TGCCGGC | -3 | 76 | 1.11 |
| CATGGCCTTGGCGGTCGATG---------**GAT**TGCCGGC | -9 | 66 | 0.96 |
| CATGGCCTTGGCGGTCGATGCGG-------**AT**TGCCGGC | -7 | 54 | 0.79 |
| CATGGCCTTGGCGGTCGATGCGG-----**GGAT**TGCCGGC | -5 | 54 | 0.79 |
| CATGGCCTTGGCGG-------------**AGGAT**TGCCGGC | -13 | 40 | 0.58 |
| CATGGCCTTGGCGGTCGA-----CCC**GAGGAT**TGCCGGC | -5 | 39 | 0.57 |
| CATGGCCTTGGCGGTCGATGCGG------**GAT**TGCCGGC | -6 | 38 | 0.55 |
| CATGGCCTTGGCGGTCGA-------C**GAGGAT**TGCCGGC | -7 | 35 | 0.51 |
| CATGGCCTTGGCGGTCGATGCGG---**GAGGAT**TGCCGGC | -3 | 32 | 0.47 |
| CATGGCCTTGGCGGTCGATGCGG---------TGCCGGC | -9 | 20 | 0.29 |
| CATGGCCTTGGCGGTCGATGC----C**GAGGAT**TGCCGGC | -4 | 18 | 0.26 |
| CATGGCCTTGGCGGTCGATGCGGC-----**GAT**TGCCGGC | -5 | 17 | 0.25 |
| CATGGCCTTGGCGGTCGAT--------**AGGAT**TGCCGGC | -8 | 17 | 0.25 |
| CATGGCCTTGGCGGTCGATGC----------**T**TGCCGGC | -10 | 14 | 0.20 |
| CATGGCCTTGGCGGTCGAT-----------**AT**TGCCGGC | -11 | 14 | 0.20 |
| CATGGCCTTGGCGGTCGA-----------**GAT**TGCCGGC | -11 | 9 | 0.13 |
| CATGGCCTTGGCGGTC---------------**T**TGCCGGC | -15 | 3 | 0.04 |
| CATGGCCTTGGCGGTC--------------**AT**TGCCGGC | -14 | 3 | 0.04 |

[1] The PAM sequence is bolded

**Supplementary Table 2.** Total cell count in each cluster (Normalized to input)

*a. TG clusters (Normalized to input)*

| | TG-1 | TG-2 | TG-3 | TG-4 | TG-5 | TG-6 | TG-7 | TG-8 | TG-9 | TG-10 | Total TG |
|---|---|---|---|---|---|---|---|---|---|---|---|
| **HSV+** | 7 | 32 | 82 | 69 | 6 | 13 | 11 | 46 | 12 | 4 | 282 |
| **AAV1+** | 15 | 51 | 88 | 110 | 37 | 11 | 33 | 29 | 29 | 18 | 421 |
| **AAV8+** | 39 | 0 | 11 | 88 | 33 | 22 | 6 | 50 | 22 | 6 | 277 |
| **AAV-PHP.S+** | 33 | 15 | 40 | 110 | 11 | 26 | 0 | 37 | 11 | 4 | 287 |
| **AAV-Rh10+** | 18 | 22 | 26 | 77 | 15 | 11 | 15 | 37 | 0 | 4 | 225 |
| **Neurons** | 328 | 230 | 505 | 406 | 197 | 136 | 97 | 177 | 130 | 113 | 2319 |

*b. SCG clusters (Normalized to input)*

| | SCG-1 | SCG-2 | SCG-3 | SCG-4 | SCG-5 | Total SCG |
|---|---|---|---|---|---|---|
| **HSV+** | 11 | 5 | 0 | 10 | 2 | 28 |
| **AAV1+** | 15 | 18 | 0 | 15 | 0 | 48 |
| **AAV8+** | 418 | 495 | 77 | 187 | 55 | 1232 |
| **AAV-PHP.S+** | 143 | 176 | 11 | 44 | 11 | 385 |
| **AAV-Rh10+** | 216 | 165 | 22 | 66 | 11 | 480 |
| **Neurons** | 616 | 871 | 211 | 208 | 135 | 2041 |

**Supplementary Table 3.** Percent neurons $HSV^{+}$ or $AAV^{+}$ in each cluster (Normalized to input)

*a. TG clusters (Normalized to input)*

| | TG-1 | TG-2 | TG-3 | TG-4 | TG-5 | TG-6 | TG-7 | TG-8 | TG-9 | TG-10 | Total TG |
|---|---|---|---|---|---|---|---|---|---|---|---|
| **HSV+** | 2.1 | 13.9 | 16.2 | 17.0 | 3.0 | 9.6 | 11.3 | 26.0 | 9.2 | 3.5 | 12.2 |
| **AAV1+** | 4.5 | 22.3 | 17.4 | 27.1 | 18.6 | 8.1 | 34.0 | 16.6 | 22.6 | 16.2 | 18.2 |
| **AAV8+** | 11.7 | 0.0 | 2.2 | 21.7 | 16.8 | 16.2 | 5.7 | 28.0 | 16.9 | 4.9 | 11.9 |
| **AAV-PHP.S+** | 10.1 | 6.4 | 8.0 | 27.1 | 5.6 | 18.9 | 0.0 | 20.7 | 8.5 | 3.2 | 12.3 |
| **AAV-Rh10+** | 5.6 | 9.6 | 5.1 | 19.0 | 7.4 | 8.1 | 15.1 | 20.7 | 0.0 | 3.2 | 9.6 |

*b. SCG clusters (Normalized to input)*

| | SCG-1 | SCG-2 | SCG-3 | SCG-4 | SCG-5 | Total SCG |
|---|---|---|---|---|---|---|
| **HSV+** | 1.8 | 0.6 | 0.0 | 4.8 | 1.5 | 1.4 |
| **AAV1+** | 2.4 | 2.1 | 0.0 | 7.1 | 0.0 | 2.3 |
| **AAV8+** | 67.9 | 56.8 | 36.5 | 89.9 | 40.7 | 60.4 |
| **AAV-PHP.S+** | 23.2 | 20.2 | 5.2 | 21.2 | 8.1 | 18.9 |
| **AAV-Rh10+** | 35.1 | 18.9 | 10.4 | 31.7 | 8.1 | 23.5 |

**Supplementary Table 4**. Percent $HSV^{+}$ $AAV^{+}$ neurons in each cluster (Normalized to input)

*a. TG clusters (Normalized to input)*

| | TG-1 | TG-2 | TG-3 | TG-4 | TG-5 | TG-6 | TG-7 | TG-8 | TG-9 | TG-10 | Total TG |
|---|---|---|---|---|---|---|---|---|---|---|---|
| **AAV1+** | 0.0 | 34.4 | 26.8 | 42.5 | 0.0 | 28.2 | 33.3 | 15.9 | 0.0 | 0.0 | 27.3 |
| **AAV8+** | 0.0 | 0.0 | 0.0 | 23.9 | 0.0 | 0.0 | 0.0 | 35.9 | 0.0 | 0.0 | 11.7 |
| **AAV-PHP.S+** | 0.0 | 0.0 | 8.9 | 26.6 | 0.0 | 0.0 | 0.0 | 15.9 | 0.0 | 0.0 | 11.7 |
| **AAV-Rh10+** | 0.0 | 11.5 | 0.0 | 21.3 | 0.0 | 56.4 | 0.0 | 8.0 | 0.0 | 0.0 | 10.4 |

*b. SCG clusters (Normalized to input)*

| | SCG-1 | SCG-2 | SCG-3 | SCG-4 | SCG-5 | Total SCG |
|---|---|---|---|---|---|---|
| **AAV1+** | 0.0 | 0.0 | 0.0 | 0.0 | 0.0 | 0.0 |
| **AAV8+** | 100.0 | 0.0 | 0.0 | 55.0 | 0.0 | 78.6 |
| **AAV-PHP.S+** | 33.3 | 0.0 | 0.0 | 36.7 | 0.0 | 26.2 |
| **AAV-Rh10+** | 66.7 | 0.0 | 0.0 | 100.0 | 0.0 | 78.6 |
