# Supplementary material for: Gene editing and elimination of latent herpes simplex virus in vivo
Source: Nat Commun. 2020 Aug 18;11:4148. doi: 10.1038/s41467-020-17936-5 (PMC7435201; doi:10.1038/s41467-020-17936-5)
Supplement: Supplementary file 2 — Reporting Summary [file 41467_2020_17936_MOESM2_ESM.pdf]

nature research

Corresponding author(s): Keith R. Jerome

Last updated by author(s): Jun 16, 2020

Reporting Summary

Nature Research wishes to improve the reproducibility of the work that we publish. This form provides structure for consistency and transparency in reporting. For further information on Nature Research policies, see Authors & Referees and the Editorial Policy Checklist .

Statistics

For all statistical analyses, confirm that the following items are present in the figure legend, table legend, main text, or Methods section.

n/a	Confirmed	
☐	☒	The exact sample size (*n*) for each experimental group/condition, given as a discrete number and unit of measurement
☐	☒	A statement on whether measurements were taken from distinct samples or whether the same sample was measured repeatedly
☐	☒	The statistical test(s) used AND whether they are one- or two-sided<br>*Only common tests should be described solely by name; describe more complex techniques in the Methods section.*
☒	☐	A description of all covariates tested
☐	☒	A description of any assumptions or corrections, such as tests of normality and adjustment for multiple comparisons
☐	☒	A full description of the statistical parameters including central tendency (e.g. means) or other basic estimates (e.g. regression coefficient) AND variation (e.g. standard deviation) or associated estimates of uncertainty (e.g. confidence intervals)
☒	☐	For null hypothesis testing, the test statistic (e.g. *F*, *t*, *r*) with confidence intervals, effect sizes, degrees of freedom and *P* value noted<br>*Give P values as exact values whenever suitable.*
☒	☐	For Bayesian analysis, information on the choice of priors and Markov chain Monte Carlo settings
☒	☐	For hierarchical and complex designs, identification of the appropriate level for tests and full reporting of outcomes
☒	☐	Estimates of effect sizes (e.g. Cohen's *d*, Pearson's *r*), indicating how they were calculated

Our web collection on statistics for biologists contains articles on many of the points above.

Software and code

Policy information about availability of computer code

Data collection	For scRNAseq Image analysis and base calling were performed using RTA Version 1.18.66.3. Sequencing reads were processed with the 10X Genomics 'Cell Ranger' v2.1.0 and with Seurat v2.3.4
Data analysis	Graphs and statistical analysis were made using GraphPad Prism version 7. Code used to process NGS data to determine the mutation rates is available at [https://github.com/proychou/TargetedMutagenesis], and code used for the scRNAseq analysis is available at [https://github.com/proychou/HSV_10x].

For manuscripts utilizing custom algorithms or software that are central to the research but not yet described in published literature, software must be made available to editors/reviewers. We strongly encourage code deposition in a community repository (e.g. GitHub). See the Nature Research guidelines for submitting code & software for further information.

Data

Policy information about availability of data

All manuscripts must include a data availability statement. This statement should provide the following information, where applicable:
- Accession codes, unique identifiers, or web links for publicly available datasets
- A list of figures that have associated raw data
- A description of any restrictions on data availability

Data Availability
The source data underlying figures 1b-d, 2b–c, 2e-f, 3b-c, 3e-f, 4b-e, 5b-c, 5e-f, 6b-k and 8b-i, and supplementary figures 1b-d,1f-h, 2b, 3b-c, 4a-i and 5b-j are provided as a Source Data file.
Single-cell RNA sequencing data is available via GEO under accession GSE151811.
Raw sequence data has been uploaded to SRA under BioProject PRJNA330548. Meganuclease sequences are Cellectis proprietary information and material.
Code availability
Code used to process NGS data to determine the mutation rates is available at [https://github.com/proychou/TargetedMutagenesis], and code used for the

scRNAseq analysis is available at [https://github.com/proychou/HSV_10x].
Data Availability
The source data underlying figures 1b-d, 2b–c, 2e-f, 3b-c, 3e-f, 4b-e, 5b-c, 5e-f, 6b-k and 8b-i, and supplementary figures 1b-d,1f-h, 2b, 3b-c, 4a-i and 5b-j are provided as a Source Data file.
Single-cell RNA sequencing data is available via GEO under accession GSE151811.
Raw sequence data has been uploaded to SRA under BioProject PRJNA330548. Meganuclease sequences are Cellectis proprietary information and material.
Code availability
Code used to process NGS data to determine the mutation rates is available at [https://github.com/proychou/TargetedMutagenesis], and code used for the scRNAseq analysis is available at [https://github.com/proychou/HSV_10x].

Field-specific reporting

Please select the one below that is the best fit for your research. If you are not sure, read the appropriate sections before making your selection.

- [x] Life sciences
- [ ] Behavioural & social sciences
- [ ] Ecological, evolutionary & environmental sciences

For a reference copy of the document with all sections, see nature.com/documents/nr-reporting-summary-flat.pdf

Life sciences study design

All studies must disclose on these points even when the disclosure is negative.

Sample size	n = 3-5 was chosen for our exploratory experiments, as to limit the animal number used but sufficient to give a meaningful result to identify the optimal parameters.<br>To determine the sample size for each experimental study, we computed the power to detect a difference in viral load at a single time point and calculated that with alpha = 0.05, n = 10-12 mice give the power to detect changes up to 0.5 logs, as seen in preliminary data.
Data exclusions	The only exclusion that was made was animals that did not show sign of infection during the acute phase as they may not become latently infected.
Replication	The findings of the studies were reproduced across the experiments using different experimental set-upsings of the studies were reproduced across the experiments using different experimental set-ups
Randomization	Allocation of animals into experimental groups was done such that animals presenting the same severity of infection during the acute phase were equally distributed across the experimental groups.
Blinding	Each collected tissue sample was assigned a reference number upon collection in order to keep investigator blinded during data collection and analysis.

Reporting for specific materials, systems and methods

We require information from authors about some types of materials, experimental systems and methods used in many studies. Here, indicate whether each material, system or method listed is relevant to your study. If you are not sure if a list item applies to your research, read the appropriate section before selecting a response.

Materials & experimental systems

n/a	Involved in the study	
x		Antibodies
	x	Eukaryotic cell lines
x		Palaeontology
	x	Animals and other organisms
x		Human research participants
x		Clinical data

Methods

n/a	Involved in the study	
x		ChIP-seq
x		Flow cytometry
x		MRI-based neuroimaging

Eukaryotic cell lines

Policy information about cell lines

Cell line source(s)	Vero cell line ATCC # CCL-81 was obtained from ATCC. HEK293 was obtained from Senator P. Wellstone UW vector core facility.
Authentication	None of the cell line used were authenticated
Mycoplasma contamination	The cell lines were not tested for mycoplasma
Commonly misidentified lines (See ICLAC register)	N/.A

Animals and other organisms

Policy information about studies involving animals; ARRIVE guidelines recommended for reporting animal research

Laboratory animals	Mus musculus, Swiss Webster, female, 6-8 weeks old
Wild animals	The study did not involve wild animals
Field-collected samples	The study did not involve samples collected from the field.
Ethics oversight	All animal procedures were approved by the Institutional Animal Care and Use Committee of the Fred Hutchinson Cancer Research Center.

Note that full information on the approval of the study protocol must also be provided in the manuscript.
